# Application of theoretical domains framework to explore the enablers and barriers to physical activity among university staff and students: a qualitative study

**DOI:** 10.1186/s12889-023-15588-w

**Published:** 2023-04-11

**Authors:** Lawrence Bismarck Ndupu, Vicki Staples, Sigrid Lipka, Mark Faghy, Nawel Bessadet, Chris Bussell

**Affiliations:** 1 School of Life Sciences, Faculty of Health and Life Sciences, Coventry University, Aliso Gingell Building, 20 Whitefrairs Street, Coventry, CV1 2DS UK; 2 Human Sciences Research Centre, University of Derby, Kedleston Road, Derby, Derbyshire, DE22 1GB UK; 3 School of Health Sciences, Faculty of Health, Education and Life Sciences, Birmingham City University, City South Campus, Westbourne Road, Edgbaston, Birmingham, B15 3TN UK

**Keywords:** University setting, COM-B model, Predictors, Content analysis, Group interview

## Abstract

**Background:**

Physical inactivity is one of the major risk factors for developing several chronic illnesses. However, despite strong evidence indicating the health benefits of physical activity, many university staff and students tend to be physically inactive. University settings provide a stable environment where behaviour change interventions can be implemented across multiple levels of change. The aim of this study is to examine the perceived barriers and enablers to physical activity among staff and students in a university setting, using the Theoretical Domains Framework (TDF), a precursor of COM-B behaviour model.

**Methods:**

This was a qualitative study carried out at a Midlands University in the United Kingdom. Eight group interviews were conducted with the sample (n = 40) consisting of 6 male and 15 female university staff (mean age = 40.5 ± 10.6 years) with different job roles (e.g., academic, administrative, cleaning and catering staff), and 12 male and 7 female students (mean age = 28.6 ± 4.7 years) at different stages of study (e.g., undergraduate, postgraduate, and international students). Interviews were audio recorded, transcribed verbatim and imported into NVivo12 software, responses were mapped using the TDF where theory-driven deductive content analysis was used for data analysis.

**Results:**

Six prominent domains were identified from the group interviews as enablers and/or barriers to physical activity among university staff and students: Environmental context and resources; intentions; social influences; knowledge; beliefs about capabilities; and social/professional role and identity. The themes emerging from the group interviews fit into all 14 domains of the TDF; however, 71% of the themes fit into the six most prominent domains.

**Conclusions:**

These findings suggest that several enablers and barriers influence university staff and students’ capability, opportunity, and motivation to engage in physical activity. This study, therefore, provides a theoretical foundation to inform the development of bespoke interventions to increase physical activity among inactive university staff and students.

**Supplementary Information:**

The online version contains supplementary material available at 10.1186/s12889-023-15588-w.

## Background

Physical inactivity has been associated with the development of several chronic non-communicable diseases (NCDs) such as coronary heart disease, stroke, obesity and diabetes, breast and colon cancer, anxiety and depression [1, 2], which contribute to an increased risk-ratio of all-cause mortality [3]. According to a study by Lee et al., physical inactivity is recognised as the fourth leading risk factor for early mortality worldwide, and attributable to more than 5 million deaths per year [4]. However, strong evidence suggests that engaging in routine physical activity (PA) can reduce the risks of developing these chronic NCDs [5] and improve overall quality of life [6]. Therefore, routine PA is vital to a person’s health and overall wellbeing [7]. The Department of Health and Social Care in the United Kingdom recommends PA as one of the most essential practices [8]. Epidemiological data indicates that adults should engage in a minimum of 150 to 300 min of moderate PA; or a minimum of 75 to 150 min of vigorous PA; or an equal combination of both weekly for considerable health benefits [9]. Currently, the updated recommendation from the Department of Health and Social Care suggests that these 150 min can be accrued in spells of any duration and/or accomplished in one or two bouts weekly while still resulting to health benefits [10]. Furthermore, it indicates that health benefits may likewise be obtained from lower amounts, intensities, and regularities of PA, especially for people with low physical fitness levels as well as for adults with disability [10].

A current health survey suggests that, in 2017, 39% (i.e., approximately 20 million people) of British adults were not meeting the Government’s guidelines for physical activity [11]. In addition, physical inactivity has been estimated to cost the UK health system around £1.2 billion annually [12]. This therefore increases the need for interventions aimed at increasing PA levels in different settings, as detailed in the World Health Organisation’s settings-based approach (SBA) [13]. The SBA proposes that health promotion activities should be carried out in different settings, including universities [13]. The prevalence of physical inactivity is also high in university settings, with 42% of staff [14] and 41% of students [15] not achieving the recommended PA levels. These high levels of physical inactivity in universities may be a justification for the promotion of health enhancing behaviours, such as PA engagement in this setting. The university is a unique setting to encourage pro-physical activity behaviours because of the large number of people working and studying there. For example, in the 2019/20 academic year, in the United Kingdom, 2,413,155 students were studying in universities across the UK, supported by over 409,055 academic staff [16]. Consequently, approximately 40% (i.e., 1,128,884) of these populations being physically inactive is a national issue that requires to be immediately addressed. The university settings also provide a captive stable population and existing social support [17], where behaviour change interventions can be implemented across multiple levels of change. Therefore, given that a large percentage of staff and students are still physically inactive and spend more than 30% of their waking hours in the university environment [17], it is now expedient to develop theory-based interventions to change behaviours towards PA in the university setting. However, in order to develop effective interventions to increase PA in university settings, it is also important to collect new data to identify the predictors of PA among staff and students, so that interventions can be appropriately linked to specific behaviour change techniques (BCTs) [18].

Numerous studies have assessed the predictors of PA among university staff and students employing quantitative or qualitative approaches, or a combined-methods approach. Deliens et al. [19] evaluated the enablers and barriers to PA and inactive behaviours amongst students in a Belgian university, indicated and found that PA was influenced by convenience, time, perceived enjoyment and self-discipline, social network (e.g., lack of social support, parental influence, and modelling), physical environment (e.g., accessibility and ease of use, time and distance required to travel, and costs of sports facilities), and macro environment (e.g. mass media and promotion). Similarly, Gómez-López et al. [20] showed that the major barriers reported for not participating in PA included time constraints, not liking PA, laziness, lack of social support, incompetence, and not seeing practicality of usefulness of PA. Martínez-Lemos et al. [21] focused on identifying the impact of the willingness to change on PA engagement among Spanish university students; they found that barriers inhibiting students from participating in PA included work obligations, time constraints and laziness. A combined-methods study by Aceijas et al. [22] suggested that lack of time, price, embarrassment, study pressure and university systems were the major barriers for PA amongst students in a UK university.

These studies taken as a whole suggest that lack of time was a consistent predictor of PA among both university staff and students, but evidence suggests that there are additional predictors of PA peculiar to university staff reported. For example, a study by Leininger & Adams [23] that examined the barriers to PA engagement among faculty, staff and administration in a university, revealed that in addition to lack of time, adhering to personal exercise schedules and inconsistencies in timetabling were major barriers. A qualitative study by Das et al. [24], examining university staff’s perceptions of barriers and advantages, suggested that (in addition to time constraints), knowledge was the main barrier preventing university staff from participating in PA. Given the pattern of findings emerging from these studies, it is important to identify predictors of PA for students and staff within the same university setting to help inform the development of interventions that are effective at increasing PA levels in diverse populations in university settings [25, 26].

The most used intervention in university settings to promote PA participation amongst staff and students is active transport (e.g., walking and cycling) [27, 28]. Other interventions that have also been successfully used to increase PA levels in university settings include standing (e.g., use of sit-stand desk) [29], decision signs and stair use (e.g., use of stair-prompt signs/posters) [30, 31], implementation intentions/action planning (e.g., planning where, when, and how to carry out an intended exercise and also on how to handle possible barriers) [32, 33], education (e.g., use of educational pamphlets, posters, email and text messages) [34, 35], supervised/organised sports and exercises [36], and incentives (e.g., use of money or vouchers) [37]. There is some evidence suggesting that multi- rather than single-component interventions targeting several predictors of PA are more effective in promoting PA in university settings [38], arguably because multi-component interventions address the individual, social, environmental, affective and cognitive factors that influence behaviour change, as outlined in the Theoretical Domains Framework (TDF) [39].

Whilst the evidence reviewed here suggests that interventions aimed at increasing PA levels among university staff and students are effective at increasing PA, especially amongst those that are inactive [38], research has generally produced mixed results [38, 40]. The heterogeneity in results may be due to differences in the countries where these interventions were carried out; and the use of diverse study design, intervention duration, range of participants and tools used to measure PA [38]. The risk of bias (primarily because of poor reporting and loss of participants during studies) and missing data about intervention components (i.e., BCTs), limit the strength of inferences concerning the most efficacious approaches and cast doubt on the evidence of efficacy, emphasising the demand for more high-quality research [38]. Furthermore, another limitation of some studies carried out in university settings to increase PA participation may be the lack of an overarching psychological theory/model to identify the predictors of PA which could be specifically targeted [41, 42]. Indeed, even though some studies described how interventions were carried out, they did not specifically mention the BCTs used, which makes it hard to improve the replicability of these studies and to understand why they were effective or ineffective [38]. Changing behaviour is complex and involves individual, interpersonal, cognitive, social, and environmental factors; therefore, the overall aim of the current study is to meet the increased demand for using psychological theories to support intervention development.

The application of theory is encouraged as an essential phase in intervention planning and evaluation, as well as in synthesising evidence, as outlined in the UK Medical Research Council’s guidance for creating and assessing multi-component interventions [43]. The past history of behaviour and the underlying determinants of change need to be appropriately identified and focused on by the intended intervention programme [44], for relevant BCTs to be selected and/or modified and personalised [45]. Theoretically identified mediators (i.e. mechanisms of action) may also be examined to obtain more understanding regarding the ways through which the intervention produces its effects [46]. Furthermore, underpinning interventions by theories offers the potential to test and validate theories, which in turn can aid the development of more efficacious theories that ultimately reinforce the optimisation of interventions [44]. Therefore, in order to appropriately understand behaviour change, all-encompassing theoretical models/theories need to be utilised to support intervention development.

On this premise, the Theoretical Domains Framework (TDF) [39] was chosen to underpin the current study which investigated the barriers and enablers to PA in a university setting. The TDF is a precursor of the COM-B (capability, opportunity, motivation and behaviour) model, which was further expanded into 14 domains used to understand behaviour and to simplify its application in complex interventions [18]. The COM-B model is the inner hub of the behaviour change wheel (BCW) that is used in complex interventions to identify those things (i.e., physical and psychological capability, social and physical opportunity, and automatic and reflective motivation) that require to be changed to accomplish an expected behaviour [18]. The COM-B model posits that for a behaviour to occur there must be an interaction between capability, opportunity and motivation [18]. Fostering capability or opportunity could reinforce motivation. Increased motivation may encourage individuals to participate in activities which would improve their capability or opportunity by changing behaviour [18]. For instance, owning a bicycle (opportunity) or having the ability to ride a bicycle (capability) may increase motivation to ride a bicycle [18].

The Theoretical Domains Framework (TDF) [47] provides a theoretical lens that can be used to assess the relevant mediating factors of behavioural matters by conceptualising models for assessing barriers to and enablers of change [18, 39], which could inform the design and development of theory-based interventions suitable to the university setting [48]. The TDF has been successfully used alone or together with the COM-B model to explore the barriers and enablers of PA in diverse contexts and settings [49–51]; however, to our knowledge, no known study has utilised these models to inform the assessment of barriers and enablers to PA in a university setting, and this remains a major gap. More research is therefore required to develop a better understanding of the enablers and barriers to PA among university staff and students, informed by the TDF. Increased knowledge of the factors that can encourage or inhibit participation in PA will help inform the design and development of novel or improved bespoke health promotion interventions that are effective at encouraging pro-physical activity behaviours in university settings. The aim of this study was therefore to use a qualitative approach for collecting new data that will help us more fully to understand barriers to and enablers of PA among university staff and students in the same university setting, using the Theoretical Domains Framework (TDF) to guide the investigation.

## Methods

### Participants

Group interview discussions were conducted to collect rich data. In order to guarantee adequate diversity of views, staff from diverse job roles (i.e., administrative, academic, cleaning and catering staff) and students from diverse nationalities and study levels (i.e., international, first year undergraduate, postgraduate taught (masters) and postgraduate research (PhD) students) at an English university were recruited between November and December 2017 using purposive sampling. Prospective participants were recruited through dissemination of posters/flyers in strategic locations on the university’s campuses (e.g., main receptions), e-mail, face-to-face contact, and advertisements through the university’s in-house communication platforms. The aim was to recruit at least six participants per group interview to be able to manage the group efficiently and encourage more open conversations amongst participants [52, 53]. Sample size was formulated based on the theoretically rooted approach which sets an initial minimum sample size of n = 40 participants, with the potential of recruiting more participants until no new information emerges at the point of data saturation, because in qualitative investigations, sample size can be difficult to pre-determine [54]. Even though data saturation was achieved after the seventh group interview, to ensure that no new information was missed, an additional group interview was carried out to determine if any new themes emerged.

### Ethical considerations

This study was approved by the Human Sciences Research Ethics Committee (HS-REC) of a Midland university in the United Kingdom (Ref no: 97-1717-LNs). A participant information sheet was sent to all participants before the study. In addition, prior to the commencement of each group interview, details about the purpose of the study, how participants’ anonymity and confidentiality would be ensured, and how the data generated would be securely stored and used were verbally communicated to the participants and provided in writing. Participants were then asked to sign an informed consent form before taking part. All methods were carried out in accordance with relevant guidelines and regulations and with the Helsinki Declaration of 1975, as revised in 2000 [55].

#### Piloting of the data collection process

A semi-structured question guide for use in the study was developed using the 14 domains of the TDF [39], in order to identify the barriers to and enablers of PA among university staff and students. The questions were carefully developed using pertinent literature [18], through an iterative process between the first author and the co-authors with ample group interview experience [56]. For example, the question for the ‘knowledge’ domain was ‘What do you know about PA? (How might you define physical activity?)’. The group interview guide was piloted amongst a group of five catering staff and five students in the university. The pilot group interviews commenced with an introductory round where the moderator was presented to the participants as a PhD researcher, after which the purpose of the group interview and ground rules were communicated to the participants. The participants were required to sign an informed written consent form and their permission sought to audio record the discussions using a Dictaphone. The participants were asked to share their opinions about what they perceived as barriers to and enablers of their engagement in PA, with the moderator refocusing the participants when they deviated from the main issues under discussion. The pilot group interviews lasted for up to 90 min [57] and did not reveal any need for major modifications either to the techniques used or constituents of the interview schedule, therefore, the results from the pilot test were incorporated in later analysis.

### Interview process

Eight group interviews (i.e., four each for staff and students) were carried out during working hours in different meeting rooms in a Midland university in the United Kingdom, at a date and time appropriate for the participants. The group interviews were expedited by the first author and an assistant moderator (i.e., a PhD student conversant with the group interview technique) whose role was only to take field notes and provide technical support during the group discussions. The same processes employed in carrying out the pilot group interview were utilised in conducting eight group interview discussion sessions. Light refreshments were provided during the group interview discussions which took 85 to 96 min (M = 89 min, SD = 4.83) for university staff and 92 to 100 min (M = 95 min, SD = 3.60) for university students. Afterwards, participants were entered in a prize draw, with the opportunity to win a £50, a £30 or a £20 Amazon Voucher [58].

### Data analysis

The data generated from the audio-recordings were transcribed verbatim in Microsoft Word using Windows Media Player. Pseudonyms were used to ensure participants’ data confidentiality [59]. The transcripts were proofread and transferred to NVivo12 Software (QSR International) where all quotes were encoded. Data was analysed using deductive qualitative content analysis [60]. Firstly, the 14 domains of the TDF were used as *a-priori* themes (i.e., the parent nodes), which formed the preliminary coding framework. Before the coding process commenced, the primary researcher (LBN) read and re-read two transcripts from the group interviews several times, to allow immersion in the data, and developed an inventory of initial codes informed by the aim of the research, evolving themes and sub-themes. A second researcher (VS) also read the same two transcripts and included some new emerging codes from this process. These researchers (BLN and VS) coded two transcripts independently and comparisons were made to ascertain the trustworthiness of the codes by evaluating divergences in applying the codes as well as standardising code application by clarifying definitions of the codes. Several meetings were held between the two researchers where disagreements or doubts were deliberated until an agreement was reached and an initial coding framework was then developed, with the emerging themes mapping to the *a-priori* themes. On one occasion where some themes were mapped to multiple domains of the TDF, and an agreement could not be reached between the two researchers, a third researcher (SL) who was conversant in utilising the TDF was asked to review them until a consensus was reached. Following this, the initial coding framework was then applied by the primary researcher (LBN) to the entire data, with emerging themes mapped to the appropriate TDF domains. Finally, relationships and differences between codes were assessed, with similar codes grouped together into main categories. This process led to better understanding of how the interviewees perceived the enablers and barriers to PA in a university setting. The findings are reported using these key themes. In order to ensure the trustworthiness of the findings, a generally applied model for trustworthiness which comprised of five conditions (i.e., conformability, transferability, credibility, dependability, and authenticity) was used [61].

During the group interviews, a question-and-answer style was used, where questions were asked one by one to obtain enough responses from the interviewees. The discussions were moderated by the primary researcher using follow up prompts for clarity and additional information were requested for when needed. Peer-briefing was used with the researcher working with a research team comprising of four academics. Every step in this study was well briefed to every member of the research team to ensure that no one had any concerns regarding the validity of the steps involved. The participants’ quotes were used to support the interpretation of the findings, to give the participants a voice in the outcomes whilst contributing to the reliability of the study. Every step of the research was well documented which helped the researcher to have a trail of all the steps that were undertaken to complete the study, in order to provide a justification for the decisions made. This is important in case another researcher wants to replicate the study. Member check was also used, where the summary of the results and interpretations were sent to the participants to cross-check if the information reflects their individual experiences. This is vital because the participants can help the researcher to minimise any mistakes, misinterpretation, or misperception of recorded data, thus validating the data. Furthermore, the researcher clearly identified how the analysis was carried out in the study, the measures that were employed to achieve the trustworthiness, and the assumptions that informed the analysis. Finally, the influence of the researcher was minimised by remaining silent when a participant was talking and providing prompts when no new information was arising from the discussions and then followed with additional questions.

## Findings and discussion

Even though several research exist to explore the enablers and barriers to PA in different settings, a limitation of previous research in university settings is the lack of use of overarching models/theories to underpin these studies. This current study addressed this issue by developing a qualitative study, which employed the Theoretical Domains Framework (TDF) to identify barriers to and enablers of PA among university staff and students (See Additional File 2), and thus to the best of our knowledge, the first to do this. In addition, although it is vital to understand the enablers and barriers to PA among university staff and students during intervention development, there are limited studies focused on assessing these among university staff compared to students, therefore studies carried out in workplaces were also utilised to support discussions involving university staff. The primary findings of this exploratory study provide interesting understandings into the multitude of factors influencing PA amongst university staff and students.

### Participants’ demographic characteristics

In total 21 university staff (i.e., academic, administrative, catering and cleaning staff) and 19 university students (i.e., PhD, international, postgraduate masters and first year undergraduate students) were interviewed. Eight participants (5 students and 3 staff) declined to participate in this study. Data saturation occurred at group interview seven, because the eighth group interview did not present any new information, and thus did not contribute to the development of new themes. The mean age of the university students was 28.6 ± 4.67 and 51% were male, and the mean age of the university staff was 40.5 ± 10.58 and 71% were female. The demographic characteristics of the participants are presented in Table 1.

**Table 1:** Demographic characteristics of group interview participants (Mean ± SD, %, n = 40)

Participants	Number of participants (M/F)	Mean age (SD)	Gender (% of females)
PhD students	5 (5/0)	31.2 ± 5.89	0
International students	6 (4/2)	32.5 ± 5.86	33.0
Postgraduate Masters students	5 (1/4)	28.8 ± 5.40	80.0
First Year Undergraduate students	3 (2/1)	21.7 ± 1.53	33.0
**Total (students)**	**19 (12/7)**	**28.6 ± 4.67**	**49.0**
Academic staff	5 (2/3)	41.4 ± 5.18	60.0
Administrative staff	5 (2/3)	36.0 ± 8.31	60.0
Cleaning staff	6 (1/5)	42.2 ± 12.66	83.0
Catering staff	5 (1/4)	42.2 ± 16.17	80.0
**Total (staff)**	**21 (6/15)**	**40.5 ± 10.58**	**71.0**
**Grand Total**	**40 (18/22)**	**34.5 ± 7.37**	**55.0**

### Enablers of and barriers to physical activity among study participants

As illustrated in Table 2, all 14 domains of the Theoretical Domains Framework (TDF) were reported as either barriers to or enablers of PA among university staff and students. However, six domains stood out: (1) environmental context and resources; (2) social influences; (3) knowledge; (4) intentions; (5) social/professional role and identity; and (6) beliefs about capabilities were identified as the prominent enablers of and barriers to PA among the university staff and students and will thus be the focus of this analysis. Focussing on these prominent six domains, which represented 71.1% of the emerging themes, is likely to reveal key insights that can shape future interventions promoting change.

**Table 2:** The coding frequencies of the domains of the Theoretical Domains Framework

Domains of the TDF	Coding frequency	% Of total
Environmental Context and Resources	321	17.0
Intentions	259	13.7
Social Influences	245	12.9
Knowledge	207	10.9
Beliefs about Capabilities	186	9.8
Social/Professional Role and Identity	128	6.8
Beliefs about Consequences	103	5.4
Skills	95	5.0
Memory, Attention and Decision Processes	78	4.1
Reinforcement	72	3.8
Emotions	64	3.4
Optimism	58	3.1
Goals	40	2.1
Behavioural Regulation	36	1.9
**Total**	**1892**	

#### Environmental context and resources

There is an onsite sports centre in the university where this study was carried out that offers a range of activities/exercises such as climbing, lawn/table tennis, basketball, netball and football, badminton, squash, Pilates, Yoga, and aerobics. The use of these facilities is available at monthly or yearly membership fees, although people are allowed to pay daily to access specific activities. However, the membership fee is only discounted for students and not for staff. This domain of the TDF was reported as both enablers and barriers to PA participation among university staff and students. All the participants in this study, regardless of their gender or age, indicated that the provision of a sports centre on campus was a motivation for them to engage in more routine PA:

‘*Well yeah, I think having the sport centre in terms of our facilities I think is great. A great facility in terms of location of the environment’* (Anita- Administrative staff).

This is consistent with previous studies [62, 63] demonstrating that the provision of a convenient and accessible onsite sports centre was one of the major environmental resources that may encourage university staff and students to engage in PA. This may be because of the proximity of the onsite sports facility and thus the ease of access. Therefore, as identified in a TDF-based analysis [18], universities, as organisations aiming to encourage their service users to be more active, should consider making sport facilities not only available but cheaper and affordable for every member, be they student or staff. However, this can be a costly investment which may hold back university stakeholders from considering it.

In addition to the provision of an onsite sports centre, which was reported as a major enabler to PA engagement amongst most university staff and students, some staff believed that the proximity of the main university campus to a park motivate them to engage in PA such as walking, especially during their lunch periods:

*‘It’s pretty good that the park is quite near if you did want to go for a wonder walk, again, only if you can fit it in around you’* (James- Administrative staff).

Likewise, some other university staff reported the proximity of some shops around the university campus as a barrier to PA engagement. They believed that this would encourage them to walk to the shops during their lunch breaks:

*I think if we were…if there were more things around the University that would get me out of the office and I know a lot of people go to park farm for lunch, kind of walk to the shops and back* (Anita- Administrative staff).

This finding supports previous research [64, 65], indicating that the proximity of university campuses to parks (i.e., green spaces) may encourage staff and students to engage in more routine PA. This is possibly because parks offer people the chance to spend ample time with colleagues, friends, or family away from the clamour and hassles in the city, bond with nature and engage in PA [66]. However, no known study has investigated the proximity of university campuses to shops as an enabler of PA engagement among staff and students, as reported in this present study. Thus, the proximity of the university campuses to green spaces as well as shops should be considered in designing interventions aimed at encouraging staff and students to engage in PA.

Some university students mentioned high-rise buildings and location of various university satellite campuses as enablers for their PA engagement. For example, one postgraduate master’s student stated that the high-rise buildings and the location of the university campuses encourages them to use the stairs more instead of the lifts:

*Erm, I think the location, because the …it’s very much, obviously I know it’s literally everything’s high, it’s not flat, all these buildings are high, so it’s a fact of, you know, I’ll always use the stairs instead of using the lift* (Michelle- Master’s student).

Similarly, an administrative staff though did not say anything about the high-rise buildings, also stated that the location of the satellite campuses was an encouragement for more routine participation in PA through walks between campuses to see students:

*So that kind of does make me think well there’s no point in…sometimes I deliberately set it up so that…because I see students over at other sites, so that I can have a walk in between from here to Britannia Mill, and that’s…I really enjoy doing that but it’s just fitting it in* (Jane- Administrative staff).

These are unique findings of this present study, as no previous research has reported high-rise buildings or locations of satellite as possible enablers of PA amongst university staff and students. This may just because of the unique nature of the university where this study was carried out, which may not be applicable to most other universities. However, these high-rise buildings could provide an opportunity for university staff and students to be more physically active by using the stairs more regularly.

Although no study has specifically assessed the effect of high-rise buildings on stair use, previous studies have shown the positive effects of placing motivational signs near lifts on stair climbing [67, 68]. Encouraging stair use may therefore be an effective and economical strategy to increase participation in PA among university staff and students. As reported in this present study, an example of how motivational signs near the lifts can encourage the use of stairs is illustrated below:

*I don’t know if this makes sense, you know the lifts where they have those labels on, that says, “Don’t use the lifts, use the stairs,”? That could be a bit of an encouragement, couldn’t it?* (Hillary- First year undergraduate student).

Furthermore, the availability of changing facilities in the sports centre and secure bicycle sheds all around the campus was reported by university staff as a major enabler of PA participation:

*‘Um… I think it’s quite good that it’s got, you know, places to lock your bikes and there’s the changing facilities in the gym’* (Wendy-Administrative staff).

The findings of this present study showed that the provision of changing facilities and safe bicycle sheds would promote more engagement in PA. This aligns with a current study suggesting that lack of resources, such as adequate changing facilities, could demotivate staff from participating in PA [62]. This may be because people may need to take a shower, freshen up and probably change their clothes after commuting to work (e.g., cycle, walk, or jog), as well as find a safe place to park their bicycles. Since this may be a critical barrier preventing university staff from opting for a healthy and active lifestyle, providing adequate changing facilities and secure bike sheds in universities may be a strategy to increase PA participation not only among staff but students as well.

Whilst onsite sports centre, high-rise buildings, location of satellite campuses, proximity of campus to a park and shops, motivational signs by lifts, and availability of changing facilities and secure bicycle sheds have been recognised as an enabler to PA engagement, the participants mentioned some specific barriers associated to resources. This included lack of time, financial constraints, inaccessibility to certain sports facilities, lack of advertisement and weather. Lack of time was a generally reported barrier to PA engagement among most of the university staff and students. Whilst most students, especially the PhD students, mentioned lack of time due to study commitments as a major barrier for not participating in PA, the university staff, particularly the administrative staff, mentioned lack of time due to work commitments:

*Like sometimes I’m in the uni till 10pm, 9pm, depending on the intensity of the work with deadlines for some publication and stuff like that. It takes so much time* (Christopher- PhD students).

*I think you know, when you have a full-time job…I don’t have kids so…but still it feels like there is just always too many things to do in a day* (Anita- Administrative staff).

Lack of time to participate in PA has been a commonly recognised barrier to PA engagement among university staff and students across various job roles and study levels, respectively. This could possibly be due to their actual or perceived workload. This current study reinforces the findings of previous research, demonstrating that lack of time was a commonly reported barrier to PA participation [62, 69]. Therefore, creating time for recreational activities in university campuses may be a strategy to encourage staff and students to participate in PA.

Financial constraint was another commonly reported barrier to PA amongst both university staff and students, regardless of their age or sexual orientation. Some of the university staff believed that even though the onsite sports centre was a big facility that offered a wide variety of exercise classes, the cost of membership was too expensive for them to afford and may therefore impact on their PA engagement:

…*but I’ve been down and it’s too expensive. I think they ought to give like a, I don’t know six weeks free or something or a month free just to encourage you. And then after that then you can decide whether it is worth the money what they’re asking. Because they do have a lot of classes down there, I think, don’t they? Because it is a big facility ain’t it? (Amy- Catering staff).*

Likewise, some students indicated that they would not be able to afford the gym membership as well as transport to the onsite sports centre because of the distance from where they live. So, this demotivates them for engaging in PA:

*I wouldn’t be able to join a gym because I can’t afford gym membership and transport to a gym, I’d have to walk, like, an hour to get to the gym and then I’ve walked an hour, I’m not doing exercise on top of that* (Martha- Master’s student).

This findings align with previous research suggesting that financial constraints was one of the commonly perceived barrier to PA engagement [19, 62]. In order to encourage staff and students to use onsite sports facilities, and thus improve their participation in PA, the university administrators should consider subsiding cost of membership.

Even though the sports centre in the university where this research was carried out is big and offers a range of exercise classes, most university staff and students reported that the inaccessibility to certain sports facilities was preventing them from participating in PA. For example, some of the academic staff believed that facilities such as a swimming pool would encourage them to participate in PA:

*Um… If they had a swimming pool that would make a difference, perhaps um… that was a real missed opportunity in my opinion.* (Catherine- Academic staff).

Similarly, most university students, especially the PhD students, indicated that the facilities in the sports centre was focused on few students. They believed that facilities should be provided to cater for all students:

*They don’t have like a way of having sports for all people, just for a few people, as Eric said, maybe 20 or 25% maximum students. If they want to increase, they have to think about completely again to what kind of facility they have and to access and, yeah (Frank- PhD students).*

The present findings are similar to previous research demonstrating that the lack of access to certain sports facilities are barriers to PA participation in a typical university setting [19, 62]. A major reason for this may be because different people may feel confident and comfortable engaging in specific types of PA, and when not available my not be motivated to participate in PA. Universities are diverse settings concerning job roles, mode and level of study and sexual orientations. Therefore, the existence of different barriers to PA participation may be apparent. The management of universities must consider this when providing sports facilities for their staff and students.

Most of the university students, especially the postgraduate master’s students reported the lack of advertisement regarding the types of sports and exercise classes offered in the sports centre as a barrier to PA engagement:

*I don’t know, erm, maybe, like, where the lecture is will depend on how many stairs I climb. And, I suppose they could have more posters about what’s available there, but I’m only at university two days a week and then the rest of it is me at home, so I don’t think it really impacts that much* (Martha- Master’s student).

On the other hand, most university staff, especially the administrative staff reported that the social timetables in the sports centre were developed with only students in mind and not staff, which is a barrier for PA participation:

*…as well and I think some of the sports…social timetables and things don’t quite fit well for staff, they are a better fit for the students. And yeah, it’s kind of geared a bit more for students than staff* (James- Administrative staff).

This present finding supports previous research regarding lack of advertisement of the available sports and exercise as being one of the reported barriers to PA participation by university students [19]. However, even though different studies have reported inconsistent work timetables as a major barrier to PA engagement among university staff [23], no study has reported social timetabling as a barrier to PA among university staff, which is a unique finding of this present study. This may be because most universities do not have onsite sports facilities and thus will not require social timetables for certain sports. Advertisement is very important, especially for new students, in order to know what is being offered in the onsite sports centre. Therefore, an orchestrated advertisement of all the sports and exercise classes offered in the university’s sports centre with a timetable is necessary, so that university staff and students can plan better to engage in their preferred sports/exercise.

Weather was another major barrier to PA that was reported by most female university staff and students, especially the international students from hotter climate regions such as Africa. Some university staff and students stated that they are likely to participate in routine PA, such as walking, but it is challenging most of the time because of the poor weather conditions in the UK. One of the international students reported engaging in lots of walking while in their home country, but hardly engage in any form of PA since coming to the UK because of the cold weather:

*The reason is this, back home in Nigeria, I used to trek from my house to the bus stop before getting a vehicle to work and then when I get to my bus stop to my workplace, I will trek again to my house… to the um… office. So, for me that was the form of exercise that I was engaging in, but when I got here the weather is so cold, so because the weather is cold, I’m not interested in going out. I just want curl up in bed* (Oluchi- International student).

This present finding reinforces with previous research regarding adverse weather conditions, such as very cold temperature and darkness during the winter period, as being one of the commonly reported barriers to PA participation [70]. In support of the findings of this present study, a systematic review by Tucker and Gilliland [71] to examine the impacts of weather on levels of PA suggested that extreme weather was a barrier to participation in PA among various populations. This may be a more significant concern, especially for females because of the safety of walking alone in the dark as a way to improve their routine PA engagement. Therefore, future interventions aimed at increasing PA in university settings should consider the effects of weather conditions. Providing indoor facilities for staff and students to use, especially during cold and rainy periods, may promote routine PA behaviours in university settings all year round.

Finally, the free bus scheme offered by the university, to ease the movement of staff and students, was one of the barriers to PA engagement reported by some university students. For example, one PhD student believed that the provision of free buses for students will discourage them walking:

*Firstly, I don’t think the university encouraging me for doing sport activity or just physical activity, but for another point of view, I noticed that Derby also does a free bus for students, it doesn’t encourage them to walk, so they’re just lazy* (Frank- PhD student).

This is a unique finding of this present study. Even though several studies have encouraged active transport (e.g., walking and cycling) in universities [28, 72], no previous research has reported the effects of free bus schemes. The fact that previous studies carried out in universities did not find free bus schemes to influence university students’ PA behaviour could be explained by the possibility that most universities may not have this scheme in place, resulting in more active transport.

Therefore, the findings of this current study and an evaluation of the wider literature show that environmental context and resources are powerful both in terms of enabling PA but also in terms of creating barriers to engaging in PA. This implies that a careful consideration of environmental factors and resources is required when developing strategies to improve PA participation among university staff and students.

#### Social influences

Apart from the physical environment, the social environment was another major determinant of PA reported by university staff and students. Social influence was commonly reported by university staff and students as both an enabler and a barrier to PA participation. Most university staff indicated that they would engage in more PA when they have their family or colleagues with them:

‘*Yes, definitely yes, with the social influence I mean, colleagues, family, I do more stuff*’ (Joseph- Academic staff).

Unlike university staff that mentioned engaging in more PA when they have their family and colleagues with them, university students reported that the would be encouraged to engage in PA when they have their family and friends with them, with no mention of influences from their colleagues/peers:

*Yeah, so friends, family um… that’s what I found with myself, so there’s a lot of encouragement between each other, and you can enjoy it more if you’re with people that you enjoy being around* (Andrew- PhD student).

Several studies have also reported the importance of family and friends support (i.e., social influences) as an enabler to PA engagement amongst university staff and students [73, 74], as found in this present study. This may be because of the supportive role of friends and family members, which may ultimately improve peoples’ self-efficacy and motivation to participate in PA [75, 76]. However, limited studies have reported the influence of colleagues on engagement in PA amongst university staff, as reported in this current study, probably because people may generally feel more comfortable exercising with people they already know very well. Therefore, it may be that the staff in this study were already very comfortable exercising with their colleagues. Universities could provide opportunities to encourage staff to exercise with their colleagues as a strategy of improving PA engagement among university staff.

On the other hand, even though most university staff and students indicated that they would engage in more PA when colleagues, friends and family are with them, others, especially the female staff, reported that family commitments (i.e., caring for the family) leaves them with no time to participate in PA:

*So, add that on to my working day, then add on going home, picking up my child, um… cooking dinner, getting her to bed, that leaves me, if I’m lucky, half an hour* (Monica- Academic staff).

The present finding reinforces previous research demonstrating that family commitments, especially childcare responsibilities, prevents some university staff from participating in PA [77]. This is probably because they spend most of their time at work and when they return home, they would want to spend more time with their family, with most of this time being spent inactive. Nevertheless, it is evident that social influences have some positive effects on PA engagement, therefore, considering ways to incorporate social support into future interventions in universities may be an effective strategy to increase staff and students’ participation in PA.

#### Knowledge

Knowledge has been associated with participation in PA in the literature [78, 79]; however, knowledge about the government’s recommended PA guidelines was generally low amongst both university staff and students. Only one postgraduate master’s student and one academic staff member were able to correctly state the recommended PA guidelines for adults:

*Yeah, with, erm, because erm, at work, we do Health Psychology Masters, so I’ve actually looked it up before and it’s something like 150 min of mild to moderate per week…with two sessions per week of stretching and then there’s something else as well. Oh yeah, like, weight, or… Something like weights* (Martha- Master’s student).

*‘Um… so, current recommendations are 30 minutes 5 times a week moderate intensity exercise’* (Mark- Academic staff).

On the contrary, most of the university staff and students could not correctly state the government’s recommended PA guidelines for adults. Most of the participants either overstated or understated the daily or weekly recommended minutes to engage in moderate-vigorous intensity PA each week:

‘*Um… I think I read as well or heard 20 minutes a day’* (Sophie catering staff).

The participants in this current study generally lacked knowledge about the recommended PA guidelines for adults, with only two participants correctly stating the PA recommendations, which is consistent with previous research [80, 81]. In support of this finding, previous studies carried out in different countries around the world also reported low awareness of PA guidelines, ranging from 4.4% in China [78] to 36.1% in the U.S. [82]. This high divergence of knowledge about PA guidelines reported globally may be due to unequal dissemination of PA recommended guidelines [81]. This finding is very important because good knowledge of PA has been shown to boost PA engagement [81]. So, it is not surprising that educational interventions have been employed to effectively increase the knowledge about PA guidelines [34]. In addition, it is also notable that a university is comprised of well-educated people that might be expected to be more knowledgeable about PA. Therefore, poor knowledge about PA, as reported amongst university staff and students in this study, should be of great concern, which needs addressing through mass dissemination and awareness programs. Awareness of PA guidelines should be encouraged more in university settings because it provides direction on the regularity, types and intensity of PA needed to gain the health benefits [79].

Even though a majority of the university staff and students did not know the recommended PA guidelines for adults, some of them were able to mention a range of benefits from engaging in routine PA. They also indicated that knowing these benefits encourages them to engage in PA more:

*The main influences I suppose are just my knowledge that it is good for me. That’s my…that’s what influences me the most, just my knowledge to do something that I ought to do* (Jane- Administrative staff).

The current findings reinforce previous research where knowledge about the benefits of participating in PA has been highlighted as one of the enablers of PA. For example, a study by Fredriksson et al., [79] examining if there was a difference in how detailed an individual’s knowledge is about the benefits of PA, suggested that individuals with more knowledge about the benefits of PA and the harmful impacts of physical inactivity were more likely to engage in PA. Therefore, health promotion efforts in universities should raise more awareness about different diseases linked to physical inactivity as a strategy to increase PA participation amongst staff and students.

Moreover, some university staff and students stated that knowing how to easily access PA initiatives provided on campus such as specific sports events will be an incentive (i.e., enabler) for them to engage in PA:

*Erm, I think knowledge would be an incentive in regard to the…if something was…if more information was provided on, say, the easily accessible nature of something, you know, more information, if there was a lot of information provided about a particular sport event or something like that, that’s an incentive for me* (Michelle- master’s student).

Sports events are conducted all year round in the university where this study was carried out, especially during inductions for new students. This finding is unique as no study has reported receiving information about how to easily access sports events in a university campus as enablers of PA, so it was difficult finding previous evidence to support this. This may be because most of these sports events are mainly focused on first year undergraduate students. Therefore, the university authorities should consider promoting these sports events as a university-wide approach to improve staff and students’ PA levels and overall wellbeing.

#### Intentions

Intention is a deliberate determination to engage in a behaviour or resolution perform it in a particular way [39]. Intention has been recognised as an important construct in commonly used health behaviour theories such as the transtheoretical model [83], theory of planned behaviour [84], social cognitive theory [85], and more recently the theoretical domains framework [39]. In this present study, the majority of university staff and students felt motivated to participate in PA to prevent the detrimental impacts of physical inactivity or to experience the beneficial effects of PA. Most participants stated affirmatively that they would love to engage in more PA:

‘*Of course, I would, I’d love to do more physical activity. Yes, most definitely’* (Jennifer- Catering staff).

*Of course, I would like to do more physical activities* (Mohammed- International student).

In addition, there were mixed feelings regarding the intentions to engage in different forms of PA, with some university staff and students clearly indicating that they want to experience new things and would participate in different forms of PA:

‘*Yes, some different activities, why not, experience something new’* (Joseph- Academic staff).

*…erm, and do, yeah, do different things. Like I’d like to do more, erm, weight things …erm, and then, yeah, I’d also like to get back into competitive sport because I used to always do team sports …erm, but then since going to university, left all those teams, so then, it’s like having to find a new team to join or whatever. Erm, so I would like to do that, and, yeah, different, different things* (Jacqueline- Master’s student).

However, some others were not keen on engaging in different types of PA, but would prefer to engage in more of what they were already doing:

*I want to do more of the same thing, referring to the gym aspect getting back to my workout routine five days a week, I want to get back to that and I want, we’re leaving out the food bit, so yes, I do want to get back in the gym and get back on that routine. As far as anything else, um, I don’t see myself doing anything else right now* (Barak- First year undergraduate student).

Although most university staff and students had the intention to engage in PA, with some even planning to engage in different types of PA or continue with what they were already doing, a few of them did not have the intentions to even engage in any form of PA. Not having a companion to engage in PA with was one of the reasons given by a catering staff for not participating in PA:

*I don’t really, I don’t really do anything. I used to go out on my pushbike a lot but I’m going to in summer because I haven’t got a dog now. My life has changed quite a bit since I lost the dog really because I used to go out a lot more when I got the dog (Amy- Catering staff).*

Intention has been established as a strong predictor of behaviour change, with previous studies reporting the association between intention and increased PA engagement [86]. In the current study, participants reported intentions as both barriers to and enablers of PA engagement, with some stating that they plan to engage in more PA by either participating in the same or new types of PA, and others stating that they do not plan to engage in more PA. Intentions have been consistently shown to be associated with behaviour, however, most of the disparity in PA was not accounted for by intentions [86]. Therefore, to understand behaviour, it is expedient not just to understand intentions, but also those factors that affect the likelihood of acting on intentions [86].

Although intentions could unquestionably have an impact on a myriad of health behaviours, the greatest intentions are often not successful in transforming into the expected behaviour [87]. For example, people sometimes set targets for PA, but subsequently find themselves discouraged or frustrated by their inability to engage in or continue participating in the PA [87]. The significance of intentions in influencing behaviours, such as PA, resulted in the development of implementation intentions [88, 89]. According to Aarts, Dijksterhuis & Midden [90], having strong intentions to perform a behaviour does not necessarily result in the performance of that behaviour. However, forming implementation intentions of how, when, and where to engage in a behaviour heightens environmental cues to act and thus enhance memory to engage in the anticipated behaviour [91]. This may be because implementation intentions mentally links expected critical situation with efficient goal-focused responses [89]. Therefore, the formation of implementation intentions of the times, locations, and days to participate in PA may be considered in interventions aimed at encouraging university staff and students to participate more in PA.

#### Social/professional role and identity

The social/professional role and identity domain of the TDF, i.e., a coherent set of behaviours and displayed personal qualities of an individual in a social or work setting [39], was reported by both university staff and students in this present study as either enablers or barriers to PA participation. For instance, the cleaning staff believed that even though all their colleagues may not be achieving the recommended PA guidelines, the nature of their work required them to be physically active and thus important in their job role:

*As I said earlier, our job involves lots of physical activity and therefore demands us to be physically active. Although, I cannot say that all my colleagues meet the recommended physical activity guidelines, taking up physical activity opportunities with my colleagues to be active is an important part of my identity as a cleaning staff* (Joe- Cleaning staff).

On the other hand, the first-year undergraduate students believed that they have been engaging in routine PA because the university sees PA as important to students and thus offer them lots of opportunities to be active. One of the key initiatives the first-year undergraduate students mentioned that the university authority was doing to keep them active includes the organisation of fairs where they are encouraged to join different clubs or societies:

*I was encouraged to start doing physical activity when I joined the university. They had this fair in the atrium with different clubs and societies for students to join. So, I strongly believe that the university sees being physically active as important for students, and this has influenced me to be active* (Donald- First year undergraduate student).

Unlike the first-year undergraduate students, the academic staff do not see engaging in PA as part of their role, because they reported that the university authority was not interested in encouraging them to be active. Even with the associated benefits of PA and decrease in absenteeism to work, excessive staff workload prevents them from engaging in any form of PA [62]. This shows that the university authority does not care if staff engage in PA or not, which is a major barrier to participating in PA:

*The university authority is not interested in encouraging the academic staff to be physically active. They just increase our workload almost on daily basis and do not really care if we engage in any form of physical activity. Since physical activity has been associated with many health benefits, as well as reduction in absenteeism from work, we should be given time to engage in some form of physical activity. However, this is not the case, as the university authority do not believe physical activity is important for us. That’s my own opinion* (Lynda- Academic staff).

Likewise, the PhD students mentioned that the university authority created more opportunities for the undergraduate students to be active through several initiatives such as sports fairs. They believed that other students were not given the same focus and thus not encouraged to be physically active:

*The university only cares about the undergraduate students. I remember them having sports fairs in the atrium for the undergraduates to encourage them to be physically active. For us, the university does not care if we are active or not. I guess that is why most of us are inactive. There is no encouragement from the university to make PhD students physically active* (Christopher- PhD students).

Previous research has discussed the lack of management support as one of the perceived barriers to PA participation [62]. In a study by Safi et al. [62], the participants, which included administrators, professional service, academics, and senior management staff, reported the management was not supporting them enough to participate in PA. This was in line with one of the findings of this present study where the academic staff also mentioned lack of management support as the perceived barrier to engaging in PA. Another finding this current study also showed that the first-year students were encouraged to engage in PA because the university management sees PA as important to them, whilst PhD students are not encouraged to participate in PA. This is a unique find as no other study has reported this yet. Therefore, further large-scale studies are needed to confirm these reported influences of social/professional role and identity on PA engagement amongst university staff and students.

Although the impact of social/professional role and identity as an enabler of or barrier to PA participation by university staff and/or students has not been widely investigated, its impact has been investigated in the PA context across a range of other populations and settings. For example, related studies which examined barriers to and enablers of PA among overweight pregnant women [49] and elderly people living with HIV [51] using the TDF, suggest that social/professional role and identity is both an enabler of and a barrier to PA engagement. Therefore, when designing interventions to promote PA engagement in a university setting, it is worth considering issues concerning the social/professional role and identity as it affects staff and students and focusing PA initiatives on all staff and students.

#### Beliefs about capabilities

The beliefs about capabilities domain of the TDF, i.e., acceptance of the truth, reality or validity about an ability, talent or facility that a person can put to constructive use [39], was reported as enabler and barrier to PA participation by university staff and students. In this present study, university staff and students had different views about their self-confidence to engage in PA. Those with self-confidence in their own functional capabilities were more likely to be active compared with those who lacked self-confidence. The majority of university staff and students believed that they were fit and could engage in any form of PA they set their mind to:

*I think, yeah, I think if I put my mind to it I could do it. I could do because I’ve not got anything wrong with me you know what I mean like, I’m fit enough, she says, um… no I’m fit enough I think for my age, so it is like yeah, I could do anything I set my mind to it or wanted to* (Anne- Academic staff).

Conversely, some university staff and students suggested lack of self-confidence or ability to engage in PA as a major barrier preventing them from participating in any form of PA. For example, an international student stated that the difficulty in participating in PA and distance to sport facilities were the major barriers preventing PA engagement, whilst an academic staff indicated that the main barrier preventing PA participation was an existing health condition:

*Um… to me I think number one, it’s difficult. Why? Initially there used to be gym very close to my house at Moorways and they closed that place. Now which means I have to come to town, you know, and from my house to town will be about 30 or 40 min to town. So, when I think about the time I have to walk or take the bus, and if I decide to stay back at uni, I don’t normally come to uni every day, so as much as I would have loved to go to gym maybe two times in the week or three times, the distance to my house that’s number two* (Titilayo- International student).

*‘I can’t do everything I want to do anymore because my lower joints won’t let me.’* (Catherine- Academic staff).

In the current study, self-efficacy (i.e., beliefs about one’s own capabilities) as outlined by the TDF was mentioned as both an enabler of and a barrier to PA engagement by university staff and students. Some participants reported that having the self-confidence (i.e., self-efficacy) to engage in PA encourages them to participate in PA. On the other hand, the lack of self-efficacy identified by some other participants as a barrier to PA engagement is of concern because self-efficacy has been recognised as a major determinant in increasing PA [92]. In support of this, a study by Pekmezi, Jennings & Marcus [93] suggested that the level of self-efficacy individuals have may significantly influence their behaviour. For example, people that believe they can successfully carry out a behaviour are more likely to carry out the behaviour [93]. Due to its significance in behaviour change, self-efficacy has been widely used in diverse health fields such as weight management, diet, alcohol consumption, sun protection, smoking and alcohol consumption [93].

Self-efficacy has also been reported to influence the types of activities people may choose to engage in, the amount of energy they spend to pursue their goals, and the levels of perseverance they exhibit regardless of obstacles, challenges, or disappointments [94]. Therefore, it is hard to dispute that choice, determination, and perseverance are not associated with the effective participation and continuation of a PA routine. Consequently, self-efficacy has been associated with intricate health behaviour, and undeniably, it has been prominently reported as a major correlate to PA participation [95]. Self-efficacy is the most commonly known psychosocial determinant of PA, thus, it is imperative to assess and if required, improve people’s self-efficacy for PA. A systematic review by Ashford et al. [96] reported some strategies that have been successfully used to improve self-efficacy for PA, and they include PA prescription (i.e., recommending the types of PA people should engage in); vicarious experience (i.e., using peer role models to carry out sessions of group PA); goal setting (i.e. setting targets for people to achieve); feedback (i.e., providing feedback by comparing a person’s performance with that of others); and tailored interventions (i.e., focusing interventions to specifics groups to change behaviour) [96]. Therefore, these strategies can be used to inform future PA interventions aimed at enhancing the self-efficacy for PA, as an effective strategy to promote PA engagement among staff and students in universities.

## Limitations

Despite the strengths of the current study that contributed novel insights into the barriers and facilitators relating to PA in a university setting based on an in-depth theory-guided exploration of the views of a range of university staff and students, some limitations need to be acknowledged. The data in this study was drawn from a sample of a medium-sized city university and thus may not be generalisable to other universities nationwide. Evidently, tertiary institutions differ widely by structure, size, area, staff and student populations, and these variations may impact on the factors that influence engagement in PA. Future studies need to examine these factors within a variety of universities in different regions. The small sample size used in this study, especially among the first-year undergraduate students, with only three individuals participating in the group interview, may be viewed as another limitation of this study which should be addressed in future studies. It is important to note that this was an exploratory study, which was not intended to gain a representative sample and group membership in itself. However, group interviews were convened to make participants feel at ease about being interviewed among other individuals like themselves. Therefore, the main area of interest was to explore factors across the groups and future studies need to establish whether individual interviews will reveal similar patterns. Previous studies have also used similar sample size [97]. Although social desirability bias may also be seen as a limitation of this study, several strategies were used to minimise it. These strategies included asking indirect questions (e.g., posing questions about the past or actions of other); providing assurances (e.g., reminding participants about confidentiality and anonymity etiquettes and assuring them that their opinions are not wrong); probing for more information (e.g., using prompts or asking follow-up questions); requesting examples or narratives (e.g., asking participants to narrate a personal experience); and prefacing some questions (e.g., recognising that all populations have challenges and that individuals have different experiences). Even with these limitations, the findings of the current study contribute to a better understanding of the factors that influence PA engagement among university staff and students, which may be subsequently used to inform the development of tailored theory-based interventions aimed at increasing PA in university settings, as well as other settings.

## Conclusion

Employing the TDF is an initial theoretical step to provide an explicit understanding of the behavioural influences in a specified context in order to conduct a concrete behavioural assessment of what requires to be altered to address unhealthy behaviours towards PA through the implementation of adequate interventions. This study provides a vital exploration of the enablers and barriers impacting on PA among university staff and students. The findings of this study suggest that having access to an onsite sports centre, social support from friends, family and colleagues, and the intention and self-efficacy to participate in PA, as well as having awareness about the recommended PA, benefits of PA, and available sports and exercise facilities may serve as enablers and contribute to university staff and students’ PA behaviour. Furthermore, lack of management support, lack of awareness about PA recommendations, time and financial constraints and family, work, and study commitments, lack of social timetabling, free bus scheme, and lack of self-efficacy to engage in PA are considered key barriers, and providing time, especially for staff to participate in PA may improve overall staff and student engagement in PA in universities. This present study identified explicit and generalisable enablers and barriers from university staff and students’ perception regarding PA participation in the university setting. The findings add to the sparse literature in terms of assessing enablers and barriers to PA among staff and students in the university setting. The current findings can inform future practices such as PA, as well as health and wellbeing-associated interventions intending to reach university staff and students. The current findings present significant and transferable results concerning university staff and students’ enablers and barriers to PA behaviour in the university. Researchers, clinicians, and intervention developers designing PA interventions for university staff and students should incorporate strategies to address these barriers.

Therefore, developing university-based interventions that target these prominent enablers and barriers has great potential of encouraging pro-physical activity behaviours among university staff and students.

## Electronic supplementary material

Below is the link to the electronic supplementary material.

Additional file 1. Group interview guide. This table illustrates the lead researchers’ questions and potential prompts developed for physical activity using the theoretical domains framework (TDF).

Additional file 2. This table provides all the enablers and barriers to physical activity among university staff and students, with description of the TDF domains, sub-themes, and sample quotes from the participants.

## Data Availability

The datasets (i.e., transcripts) generated during the current study are available in the Mendeley Data repository, https://data.mendeley.com/datasets/tz9jj7pzm9. Other datasets used and/or analysed during this study are available from the corresponding author on reasonable request.

## List of Abbreviations

NCDs: Non-communicable diseases
PA: Physical activity
TDF: Theoretical domains framework
UK: United Kingdom
