# Supplementary material for: Application of theoretical domains framework to explore the enablers and barriers to physical activity among university staff and students: a qualitative study
Source: BMC Public Health. 2023 Apr 11;23:670. doi: 10.1186/s12889-023-15588-w (PMC10088198; doi:10.1186/s12889-023-15588-w)
Supplement: Supplementary file 1 — Additional file 1. Group interview guide. This table illustrates the lead researchers’ questions and potential prompts developed for physical activity using the theoretical domains framework (TDF). [file 12889_2023_15588_MOESM1_ESM.pdf]

Group Interview question guide

**Domains of the Theoretical Domains Framework (TDF)**	**Questions**
Knowledge	What do you know about physical activity? How might you define physical activity? Is anybody aware about the recommended guidelines for physical activity?
Beliefs about consequences	Thinking about what we have discussed, do you think you achieve the recommended physical activity levels? Why do you think that they recommend you engage in physical activity to that level?
Memory, attention and decision processes	What do you think are the best ways for you to become physically active? (Prompt: what do you think are the best forms of physical activity? How will you go about increasing your activity?). Do you think students/staff engage in the recommended levels? If not, why?
Intentions	So, do you engage in any form of physical activity? And if so, what sort of physical activity do you engage in? How would you go about increasing your physical activity?
Beliefs about capabilities	For those of you that do physical activity, why do you do it? For those of you who don't engage in physical activity how confident are you that engaging in physical activity is something you can do? Are there anything that are really preventing you from being more active?
Goals	For those of you that engage in physical activity, do you have any specific goals in mind when engaging in physical activity?
Behavioural Regulation	How do you know how much physical activity you have done?
Emotion	Thinking about when you do or when you have done physical activity how did or how does it make you feel?
Skills	How difficult or easy is it for you to engage in physical activity? (Prompt: If you want to engage in physical activity, do you have the skills to do it?).
Optimism	Would anyone in this room like to do more physical activity? Do you want to do more of the same thing or different things? What type of things would you like to do? Why these things? What is currently preventing you from getting involved in those different types of activities you mentioned earlier?

Social/professional role and identity	What do you think are those things that the University is doing to engage staff/students in physical activity? So in essence, do you think there are enough opportunities for staff and students here at the university to engage in physical activity?
Social Influences	What would you say are the main influences on your decision to engage in physical activity? (Prompt: Do you engage in more or less physical activity when your friends, family or colleagues are with you?)
Reinforcement	In your view, what are those incentives that will encourage or support you to engage in physical activity? (Prompt: It may be either financial, it could be money, gift vouchers or non-financial. It could be appreciation, praise, competition, knowledge, or goal achievements, etc.).
Environmental context and resources	Thinking about the university setting, are there any aspects of the university environment that would influence whether or not you engage in physical activity?
Ending	Of all the things we’ve discussed here today, what is the most important to you? Are there other things related to physical activity at the university that I didn’t ask you about that are important for me to know? Would you want to be contacted to take part in the behaviour change intervention that would be designed based on the outcomes of this group interview? If you want to participate, you need to provide your preferred mode of communication; so, how do you want me to contact you? Is it through email or phone?
