# Supplementary material for: Application of theoretical domains framework to explore the enablers and barriers to physical activity among university staff and students: a qualitative study
Source: BMC Public Health. 2023 Apr 11;23:670. doi: 10.1186/s12889-023-15588-w (PMC10088198; doi:10.1186/s12889-023-15588-w)
Supplement: Supplementary file 2 — Additional file 2. This table provides all the enablers and barriers to physical activity among university staff and students, with description of the TDF domains, sub-themes, and sample quotes from the participants. [file 12889_2023_15588_MOESM2_ESM.pdf]

Enablers and barriers to physical activity among university staff and students

Table 1: Enablers to physical activity among university staff and students

TDF domains (a priori themes)	Subthemes	Participants		Sample quote
		Staff	PhD students	
**Knowledge** (an awareness of the existence of something)	• Awareness of the recommended physical activity guidelines	√	√	*Yeah, with, erm, because erm, at work, we do Health Psychology Masters, so I've actually looked it up before and it's something like 150 minutes of mild to moderate per week…with two sessions per week of stretching and then there's something else as well. Oh yeah, like, weight, or… Something like weights (Martha- Master's student).*<br><br>*Um… so, current recommendations are 30 minutes 5 times a week moderate intensity exercise (Mark- Academic staff).*
	• Awareness of available sports/exercise facilities	-	√	*Erm, I think knowledge would be an incentive in regards to the…if something was…if more information was provided on, say, the easily accessible nature of something, you know, more information, if there was a lot of information provided about a particular sport event or something like that, that's an incentive for me, because, I think, it's… it's that knowledge of it's easy to do, I can do this, it's okay (Michelle- master's student).*
	• Awareness of the benefits of physical activity	√	-	*The main influences I suppose are just my knowledge that it is good for me. That's my…that's what influences me the most, just my knowledge to do something that I ought to do (Jane- Administrative staff)*
**Social influences** (those interpersonal processes that can cause people to change their thoughts, feelings and behaviours)	• Acknowledged support from family, friends, and colleagues	√	√	*Yes, definitely yes, with the social influence I mean, colleagues, family, I do more stuff (Joseph- Academic staff).*<br><br>*Yeah, so friends, family um… that's what I found with myself, so there's a lot of encouragement between each other, and you can enjoy it more if you're with people that you enjoy being around (Andrew- PhD student).*

Sensitivity: Internal

**Reinforcement** (Increasing the probability of a response by arranging a dependent relationship, or contingency, between the response and a given stimulus)	• Financial incentives:<br>➢ Free/cheaper exercise classes	√	√	*It's like most of us finish at…well, most of the cleaners finish at nine o'clock now if they had an incentive where if you went to the gym from nine till half past ten say… then you could have it for a quarter of the price or for free. People would, even though they've got other jobs, they… they could make, you know, alterations to their life to be able to put that in their goal and go and do the other job (Kate- cleaning staff).*
	➢ Gift voucher	-	√	*Yeah. I mean, just if it was, not even like a financial incentive, just it was free or the transport there was free or something that would be enough than a financial incentive, and if it was something that was more, like I said, it isn't really expected in my family, if it was something that was more expected and it was a bit, why aren't you doing that, then I think that might change it (Martha- Master's student).*
	➢ Money	√	√	*Yeah, I think, erm, the first option, yeah, the financial factor or, erm, maybe a gift, like voucher and a voucher is given to me to go and do a physical activity for free. Yeah, why not?* (Audrey- Master's student).<br><br>*Um… I would definitely do it if someone was paying me a fiver, definitely! Um… the competition bit for me, only when I am there. It wouldn't get me there, but once I am there* (Jane- Administrative staff).<br><br>It's um… so I would rather go for tangible rewards, maybe financial (Ali- International student).
	➢ Pay as you go	√	-	*Pay as you go, yeah, pay as you go* (Kate- Cleaning staff).
	• Non-financial incentives<br>➢ Appreciation and praise	√	√	*I think to me, it's kind of strange. Financial reward might not encourage me, you know, sometimes you say that you would go and do this if you are given money. I might not even do it. I think mine would be kind of appreciation. Ah, you're looking good, you know, like those kind of things* (Titilayo- International student).
	➢ Recognition	√	-	*I think it's um… self-satisfaction really. It's what you've done and then when people sort of like think, Oh, say to you, Oh, you look good and that, and you're… you're doing something, yeah, exercise* (Aby- Cleaning staff).<br><br>*Um… so yeah, some recognition I think could be a good… could be a good incentive for me as well* (Anita- Administrative staff).
	➢ Health benefits	√	√	*Well, I guess health and I want to be more fit than I am now, because I'm not* (Joseph- Academic staff).<br><br>*…my medical assessment like if medically I know that am gaining this thing medically and my doctor says that, okay you've lost so much weight, your heart is good because you're doing more exercise, that would encourage me than financial aspect of it* (Titilayo- International student).
	➢ Competition	√	√	*I think um… I think myself, competition I think is a good incentive as in you know, not doing a sport competitively but I like um… yeah, I like competition so, you know, like for example, I*

Sensitivity: Internal

				know we do these tower challenges or something, you know, I would, you know… I think I'd like to be the one who does it in less time (Anita- Administrative staff)<br><br>*Yeah, even I agree with them, I don't think the money is the main purpose for that kind of thing, but the competition, when you find someone that um… just the competition, this is going, let's say, the waiting to go and increase your skills in this kind of stuff, so the motivation for that, competition in my opinion is the point* (Elvis- PhD student).
	➢ Goal achievement	√	√	*Um… the only thing I can think of is to the incentive I suppose is that you get a result that you… you want if you work at it, it might be lose your weight* (Laura- Cleaning staff)<br>*Um, for me I'd say competition and goal achievement* (Hillary- first year undergraduate student).<br><br>Um… the only thing I can think of is to the incentive I suppose is that you get a result that you… you want if you work at it, it might be lose your weight (Laura)
	➢ Intrinsic motivation	√	-	*They're all internal motivators, I wouldn't be motivated by a gift or financial, it's what I want to do when I want to do it* (Mark- Academic staff).
	➢ Social factors	√	√	*Yeah, I don't think money would encourage me either. I think sort of social things encourage me more, like if you are doing something together with friends, aiming to get fit together* (Wendy- Administrative staff)<br><br>*It should always come from within really and be like a, you know, motivation, or um… assistance from friends, or maybe like a support group, or maybe a group of people who are interested in wanting to do more exercise and they don't know how to, could get together and support each other to do that* (Andrew- PhD student).
**Emotion** (A complex reaction pattern, involving experiential, behavioural, and psychological elements, by which the individual attempts to deal with a personally significant matter or event)	• Feel happy	√	√	*Oh, start with me. Well, it produces happy hormones doesn't it? So, you do feel better for it... your physical wellbeing* (Diana- Cleaning staff).<br><br>*Um… I feel um… very light after doing physical activity and um… obviously it makes you feel good that you have done something, and so obviously, yes, I feel happy after it*. (Eric- PhD students).
	• Relieves stress	√	-	*Yeah, I think in the gym I don't enjoy it at all um… and um… but I try and use it to work off any angst or stress. Um… it's, it's quite a good psychological processing tool really. Um… and… and I get… and afterwards I think…not immediately afterwards, because afterwards you are hot and sweaty and actually feel really uncomfortable um… but you get a bit of an adrenaline rush and feel… feel quite um… alive really afterwards* (Peter- administrative staff).<br><br>*For me whenever I hear about any incident. Mayor's death brought a massive change for me. Um… a young guy died back home with a heart attack couple of days ago that… with these*

Sensitivity: Internal

	• Fear of death	-	√	*kinds of things, I… I usually start thinking about… I got to be more active, I shouldn't drink alcohol, I should do this, I should go to gym on every day, I should make myself more accessible, available to get engaged in this* (Ali- International student).
**Intentions** (A conscious decision to perform a behaviour or a resolve to act in a certain way)	• Plans to engage in more physical activity	√	√	*Of course, I would like to do more physical activities* (Mohammed- International student).<br><br>*Of course, I would, I'd love to do more physical activity. Yes, most* definitely (Jennifer- Catering staff).
	• Plans to engage more of the same or different types of activities	√	√	*Yes, some different activities, why not, experience something new* (Joseph- Academic staff)<br><br>*…erm, and do, yeah, do different things. Like I'd like to do more, erm, weight things …erm, and then, yeah, I'd also like to get back into competitive sport because I used to always do team sports …erm, but then since going to university, left all those teams, so then, it's like having to find a new team to join or whatever. Erm, so I would like to do that, and, yeah, different, different things* (Jacqueline- Master's student).<br><br>*Well, I'm happy doing what I am doing now. I would like to do more, but with my health and everything I might not be able to* (Jessica- Cleaning staff).<br><br>*I want to do more of the same thing, referring to the gym aspect getting back to my workout routine five days a week, I want to get back to that and I want, we're leaving out the food bit, so yes, I do want to get back in the gym and get back on that routine. As far as anything else, um, I don't see myself doing anything else right now* (Barak- First year undergraduate student).
	• Intrinsic motivation	√	-	*They're all internal motivators, I wouldn't be motivated by a gift or financial, it's what I want to do when I want to do it* (Mark- Academic staff).
**Skills** (an ability or proficiency acquired through practice)	• Have the skills to engage in physical activity	√	√	Yeah, I think I have all the skills I need to go for the sports I want. I was a competent horse rider, hockey player, tennis, squash, badminton, table tennis, so… swimming, so all the sports I enjoy doing I can already do because I did them because I was really, really um… sporty when I was younger (Monica- Academic staff).<br><br>Yes, I think I do have skills because, my point is when I was in my twenties, I used to play semi-professional cricket, so I think obviously I do have the skill to do any sort of physical activity (Eric- PhD student).<br><br>I have the skills, as I say, I love playing squash, and I'm very good at it (Bill- Master's student).

Sensitivity: Internal

**Behavioural Regulation** (anything aimed at managing or changing objectively observed or measured actions)	• Monitoring of physical activity done<br>➢ Use of Phone apps	√	√	…and sometimes because with iPhone when… if I go to gym and I put my iPhone very close to me, sometimes I put it… I attach it to myself. At the end of it it's going to come up with… because I have this thing on my iPhone that will tell you how many calories I have, you know, I have burnt. So, with my iPhone I keep tracks on how many, you know, how um… the progress of my physical activities (Titilayo- International student).<br><br>*Um… my step counter on the phone (Laura- Cleaning staff).*
	➢ Use of Fitbit	√	√	*Yeah, so I know how much I walk a week, the distance and how long it takes to get to and from work. I have got a little Fit Bit thing and it kind of emails you and tells you what you have done and.... (James- Administrative staff)*<br><br>*…but I personally have a Fitbit, so that um… tracks what I've done, so um… it's a… it's… it's not a great measure but you can sort of compare it to different days to see if you've been more active on certain days and you can sometimes the encouragement of competition with members of your family or friends that have it can increase your fitness (Andrew- PhD students).*
	➢ Use of Self-assessment	√	√	*Similar things. I know roughly how long the walk to school takes and I do that five times a week, and I know how long the cycle takes to work and I just base it on that. I don't have a Fit Bit or anything like that. I just take it from that (Wendy- Administrative staff).*<br><br>*The same, you feel it, for example, after just biking 30 or 40 minutes you can already feel it that you're biking since quite a long time, and if you're going climbing after 2 hours, you're feeling every muscle in your arm and your hands, so definitely you just feel it (Frank- PhD student).*
	➢ Setting of targets	√	-	*Because I set a certain amount of – with regards to exercise, I have a target and when I've achieved it, then I'm pretty satisfied (Joe- Cleaning staff).*
	➢ Use of training diary	√	-	*Eh… I keep a training diary, so for that aspect I would know how much I've done or need to do (Mark- Academic staff).*
**Goals** (mental representations of outcomes or end states that a person desires to attain)	• Weight loss	√	√	*Mine has always been to lose weight. If I… when I do my exercises, it is always to lose weight* (Jennifer- Catering Staff).<br><br>*Yes, to reduce my belly fat. Um… to reduce [laughs] my weight in general, yea, and most importantly just because it gives me very good feeling* (Ali- International student)).
	• Health and fitness	√	√	*Um… I think I have a broad goal of quality of life and um… and that involves keeping as fit as possible within the parameters of, my parameters, and, and staying healthy, um… because my*

Sensitivity: Internal

	• Personal improvement<br><br>• Target achievement	√<br><br>√	-<br><br>√	*concern is that, you know, our health service isn't going to be able to help me, so I have to help myself* (Lynda- Academic staff).<br><br>*Erm, well, I used to be really fit and then when I…in my first of University, I stopped doing any sort of sport, so my fitness went right down, so now, it's trying to get fitter again …erm, and me and my family are doing the Coast to Coast cycle ride …erm, at the end of July, so I'm aiming towards being at a good enough level to be able to complete that. Erm, yeah* (Jacqueline- Master's student).<br><br>*I guess, most of mine are fairly intrinsic around performing in my sport. Eh… some of that, there's kind of an underlying thing of, I work with quite a lot of high-level athletes that there is a sense that I want to be able to competently demonstrate things, but also that I want to be seen as someone that could be, eh… I guess, practice what you preach sort of thing* (Mark- Academic staff).<br><br>*I've setup my daily step target for 9,000 steps a day, so that's my kind of aim I want to achieve* (Oliver- International student).<br><br>*Um… I think with…when I run, there is a little bit of competitiveness in that bit as well because I do want to increase the time that I do each time. So, I have a goal of trying to…you know I am not happy if I don't do it below a certain time. Well not… not happy but I just want it* (Jane- Administrative staff).
**Beliefs about Consequences** (Acceptance of the truth, reality, or validity about outcomes of a behaviour in a given situation)	• Health benefits<br><br>• Economic implications<br><br>• Relieves stress<br><br>• Improvement of sleep quality	√<br><br>√<br><br>√<br><br>√	√<br><br>-<br><br>-<br><br>√	*Well, it is about raising your heart… your heartbeat, um… and um… and that helps to um…I mean that will get your heart working, it burns off the calories um… so you are fitter* (Peter- Administrative staff).<br><br>*So, I think it's important for, you know, your overall organs, your heart, your muscles and er things like that* (Barak- First year student).<br><br>*…but it's also about um… the economics of it because um… it costs the country a lot of money to deal with folks who have um… heart disease and generally poor physical health* (Peter- Administrative staff).<br><br>*…and minimise um… costs on the NHS for things like heart disease or obesity* (Wendy- Administrative staff)<br><br>*Yeah, I think in the gym I don't enjoy it at all um… and um… but I try and use it to work off any angst or stress. Um… it's, it's quite a good psychological processing tool really. Um… and… and I get… and afterwards I think…not immediately afterwards, because afterwards you are hot and sweaty and actually feel really uncomfortable um… but you get a bit of an adrenaline rush and feel… feel quite um… alive really afterwards (Peter- Administrative staff).*<br><br>*…and you get a better sleep too. I get a real good sleep* (Barak- First year student).<br><br>*Yeah, I feel more able to focus, as I said before, um… or I can also feel more able to rest, so to sleep better if I've engaged, which is quite good* (Lynda- Academic staff).

Sensitivity: Internal

	• Feel happy, better and good<br><br>• Feel more powerful, refreshed, focused and relaxed<br><br>• Boosts confidence	√<br><br>√<br><br>-	√<br><br>√<br><br>√	*So, you feel good, you feel… you're happy that you've been able to do something, so for me I'm like okay at least that walk is for a week at least I have done something now in one week* (Oluchi- International student)<br><br>*Well, it is good ain't it to be active, I think you feel better when you're healthier. You definitely, it definitely gives you a feel-good factor about yourself* (Amy- Catering staff)<br><br>*Erm, I feel refreshed, um, relaxed* (Hillary- First year undergraduate).<br><br>*Yeah, more focussed, more powerful, because I want mostly to play table tennis, so I want it to be yes, back in the game, yeah* (Joseph- Academic staff).<br><br>*But psychologically, your kind of um… feel confident if you go to gym on time or if you try to do physical activities on time, especially for women. For me it boosts my confidence* (Titilayo- International student).
**Beliefs about Capabilities** (Acceptance of the truth, reality or validity about an ability, talent or facility that a person can put to constructive use)	• Self-efficacy to engage in physical activity	√	√	I think, yeah, I think if I put my mind to it I could do it. I could do because I've not got anything wrong with me you know what I mean like, I'm fit enough, she says, um… no I'm fit enough I think for my age, so it is like yeah, I could do anything I set my mind to it or wanted to (Anne- Academic staff).<br><br>Erm, yeah, I think it's easy because I do have the time, like I don't work as much as other people. Erm, I live next to a really good, like, bus links, or my gyms like a ten-minute run away …erm, so I have like all the resources and time. Erm and I know that it makes me feel good, so I have the motivation, so that's, I think that's why it's easy (Jacqueline- Master's student).
**Environmental Context and Resources** (Any circumstance of a person's situation or environment that discourages or encourages the development of skills and abilities, independence, social competence and adaptive behaviours).	• Aspects of the university environment that encourage physical activity:<br>➢ Provision of the sports centre.<br><br>➢ High-rise buildings and location of campuses<br><br>➢ Proximity of university to shops and park	√<br><br>√<br><br>√	-<br><br>√<br><br>-	*Well yeah, I think having the sport centre in terms of our estate I think is great. A great facility in terms of location of the environment (Anita- Administrative staff)*<br><br>*Erm, I think the location, because the …it's very much, obviously I know it's literally everything's high, it's not flat, all these buildings are high, so it's a fact of, you know, I'll always use the stairs instead of using the lift (Michelle- Master's student).*<br><br>*So that kind of does make me think well there's no point in…sometimes I deliberately set it up so that…because I see students over at other sites, so that I can have a walk in between from here to Britannia Mill, and that's…I really enjoy doing that but it's just fitting it in (Jane- Administrative staff).*<br><br>*I think if we were…if there were more things around the University that would get me out of the office and I know a lot of people go to park farm for lunch, kind of walk to the shops and back (Anita- Administrative staff)*

Sensitivity: Internal

				It's pretty good that the park is quite near if you did want to go for a wonder walk, again, only if you can fit it in around you (James- Administrative staff)
	➢ Changing facilities and secure bike sheds	√	-	*Um… I think it's quite good that it's got, you know, places to lock your bikes and there's the changing facilities in the gym (Wendy-Administrative staff).*
	➢ Motivational posters by the lifts	√	√	*I think the lift one, because I remember there was a time when I wanted to… I think we were having that teaching… that teaching something that we were doing, and I was running late or... I wanted to climb the… [Laughs], I wanted to go inside the lift then I saw that noticeboard that you could encourage yourself by climbing and I ended up climbing [laughs], so I think that one is good (Titilayo- International student).*<br><br>*I don't know if this makes sense, you know the lifts where they have those labels on, that says, "Don't use the lifts, use the stairs,"? That could be a bit of an encouragement, couldn't it? (Hillary- First year undergraduate student)*
	➢ Advertisements in university buses	-	√	*That's a good… and the advertisements in the bus as well, because sometimes when you look at the distance, especially during new session and they advertise that if you're a student, if you register at so, so time, so when you look at it you will start to think what if I do this in the uni, you know, it will pay off because I will be in uni after school, after my lecture or after everything you go back and do the exercise and go back home. I think that advert is good as well (Titilayo- International student).*
	• Opportunities to do physical activity in the university:<br>➢ Physical activity initiatives	√	√	*I think this kind of um… even yesterday we were sitting in our office, there is… two girls come to our office just to give us some brochures that… to encourage us to go to gym with some details inside, I think. Um… this kind of thing arranged by the university, yeah, these kinds of things that maybe motivate us or are good things to go to, to do more and more now, like more training or more physical activities (Elvis- PhD student).*<br><br>*No, a few…sometimes they do um… running up the towers don't they? They have all the people who want to do it, you can run up every single tower in the university, and the first one who completes it gets a gym pass. They do it and they get a gym pass free for a month. So that gets not a lot, I don't know how many people actually do it, but… but quite a lot of people do it and whoever wins does get something for it and the gym (Aby- Cleaning staff).*
**Social/Professional Role and Identity** (a coherent set of behaviours and displayed personal qualities of an individual in a social or working setting)	• Importance of physical activity in participants course/job role	√	√	*As a cleaning staff, our daily work involves lots of physical activity and I think it is essentially necessary to be physically active to perform well as a cleaner. I mean cleaners are expected to be physically active, ain't we? (Joe- Cleaning staff).*<br><br>*I was encouraged to start doing physical activity when I joined the university. They had this fair in the atrium with different clubs and societies for students to join. So, I strongly believe that the*

Sensitivity: Internal

	• Opportunities to be physically active	√	-	*university sees being physically active as important for students, and this has influenced me to active (Donald- First year undergraduate student).*<br><br>*As I said earlier, our job involves lots of physical activity and therefore demands us to be physically active. Although, I cannot say that all my colleagues meet the recommended physical activity guidelines, taking up physical activity opportunities with my colleagues to be active is an important part of my identity as a cleaning staff (Joe- Cleaning staff).*
**Optimism** (the confidence that things will happen for the best or that desired goals will be attained)	• Confidence of the inactive to engage in physical activity	√	√	*Yes, I think obviously, I have the confidence to engage in physical activity (Eric- PhD student).*<br><br>*Well, I could do it yeah. I could, I'm really confident I could do it yeah. It wouldn't hold me back (Anne- Catering staff).*

√= mentioned by at least one administrative staff or students
-= not mentioned by any administrative staff or student
NB: Pseudonyms were used for all the participants to protect their identity.

Sensitivity: Internal

Table 2: Barriers to physical activity among university staff and PhD students

TDF domains (a priori themes)	Subthemes	Participants		Sample quote
		**Staff**	**PhD students**	
**Knowledge** (an awareness of the existence of something)	• Lack awareness of recommended physical activity guidelines	√	√	*I think its 3 hours a week of intensive activity. There is also for moderate activity, which should be longer, probably 1 hour a day (Richard- international student).*<br><br>*Um… I think I read as well or heard 20 minutes a day (Sophie- catering staff).*
**Social Influences** (those interpersonal processes that can cause people to change their thoughts, feelings and behaviours)	• Family commitments	√	√	*…my research and time for family, so there is no time left (Christophe- PhD student).*<br><br>*So, add that on to my working day, then add on going home, picking up my child, um… cooking dinner, getting her to bed, that leaves me, if I'm lucky, half an hour (Monica- Academic staff)*
**Emotion** (A complex reaction pattern, involving experiential, behavioural, and psychological elements, by which the individual attempts to deal with a personally significant matter or event)	• Confusion about recommended physical activity guidelines	√	-	*Yeah, you do read different things though, don't you? One month they're telling you one thing and then another month they're telling you something else in magazines and stuff. Or don't they?* (Amy- Catering staff).<br><br>*We're supposed to do it and they keep changing the things about how much you're meant to do and, you know, there was all this thing about, oh, you're meant to do 20 minutes a day, and then there was this so many hours a week, and there was this thing where, oh, 5 minutes a day is actually enough so long as it's really intense 5 minutes. So, they keep changing, they can't make their minds up, I don't think they know what they're talking about half the time* (Catherine- Academic staff).
	• Fear factor	-	√	*…but the interesting thing about that is, erm, because of how fit you have to be to play squash, because I haven't played for so many months now, there's a fear factor as well (Bill- Master's student).*<br><br>*"I don't know, because I just, I don't like the feeling of it, I don't like the, the moving and the breathlessness, because I think I panic, because I assume it's the asthma" (Martha- Master's student).*
**Intentions** (A conscious decision to perform a behaviour or a resolve to act in a certain way)	• No plans to engage in physical activity	√	√	*I don't really, I don't really do anything. I used to go out on my pushbike a lot but I'm going to in summer because I haven't got a dog now. My life has changed quite a bit since I lost the dog really because I used to go out a lot more when I got the dog* (Amy- Catering staff).<br><br>*I'm the odd one out. I'm not engaging in any physical activity and am not doing anything. It's just once a while that I do the trekking to, you know, city centre and back. I'm supposed to be going out every day to do like 30 minutes of... but I don't* (Oluchi- International student)<br><br>*Erm, well I know that I should do more, and I do think about doing more. Erm, in terms of trying different things, well I don't do anything, so everything's different* (Martha- Master's student)
		-	√	

Sensitivity: Internal

	• Do not plan to do more physical activity			*I don't know, because I just, I don't like the feeling of it, I don't like the, the moving and the breathlessness, because I think I panic, because I assume it's the asthma* (Martha- Master's student).
**Skills** (an ability or proficiency acquired through practice)	• Lack the skills to engage in physical activity	√	√	*I love swimming, but I don't have the skill so… and am really afraid that I don't want to start swimming without being trained, and I have tried my best to get myself trained like going to the council and they said they are fully booked up; I am number 65 on the waiting list (Titilayo- International student).*<br><br>*Mhmm… I don't have the skill, and I don't even know what to engage in (Oluchi- International student)*<br><br>*It is me because not that interested in... been…. Oh no, well I'm, I cannot swim, so I don't go swimming and kayaking would just terrify me. Canoeing and all that you know like I don't like heights. So, no sorry but I'm quite happy to sit in the house (Anne- Catering staff).*
**Memory, Attention and Decision Process** (the ability to retain information, focus selectively on aspects of the environment and choose between two or more alternatives)	• Difficulty in justifying time for physical activity<br><br>• Forget planned physical activity	√<br><br>√	√<br><br>√	*…so, I think there are things the university can, you know, not necessarily the sports centre I think it's also a matter of culture around staff wellbeing and it's true, the social timetable…I was interested in one and it just was like at 4 o'clock and I am at work at 4 so I couldn't do it (Anita- Administrative staff).*<br><br>*I have so much going on at work and at home which makes it hard for me to engage in any form of Physical activity. The truth is that there is no time to actually do all the activities I would love to do, if that makes sense (Anne- Academic staff)*<br><br>*Um, I don't really, um, I've not really done much while I've been at Uni, like, I keep meaning to join the gym here, I want to join, like, and do my sports while I'm here, but I just haven't got round to doing it, I'm too busy (Donald- First year undergraduate student).*<br><br>*I am working part-time and schooling at the same time, so it is about prioritising my time to do some form of physical activity. However, it is very difficult to justify time for physical activity. I really want to engage in some form of physical activity, but it is hard to find the time (Ali- PhD student).*<br><br>*On several occasions, I have planned to engage in physical activity, but due to my lab work and other study related commitments, I just forget to engage in it. I always feel bad afterwards, but this is a major barrier that prevents me from being active (Elvis-PhD student).*<br><br>*Seriously, schoolwork could be very challenging that you can genuinely forget to do physical activity that you had earlier planned to do. This has happened to me several times, especially when I have assignment deadlines to meet. You are just so busy, and every other thing is secondary (Bill- Master student).*<br><br>*Sometimes I had planned to go to the sports centre to play social badminton but forget to go because am too busy working or attending one meeting or the other. The university does not*

Sensitivity: Internal

				encourage us the admin staff to engage in physical activity. That is the truth (Wendy- Administrative staff)
	• Difficulty in deciding physical activity to engage in	√	√	*They have so many sports and exercise classes going on in the sports centre that makes it difficult for me to choose the ones to do (Joe- Cleaning staff)*<br><br>*The other day, I went with some of my co-workers to the sports centre to see exercise sessions we could go together, but they had so many which made it difficult for us decide on the exercise classes to join. If they had a swimming pool it would have been an easy choice for us because we all love swimming, don't we? (Anne- Catering staff)*<br><br>*I went to the sports centre the other day to look for an activity to start doing but was overwhelmed by many options of sports and exercises that I can do and up till now am still trying to decide which activity to do (Martha- Master's student).*<br><br>*Me that is very lazy. Um… one of my friends and I went to the sport centre to sign up for an exercise class and it was difficult making up our minds on which ones to do, because they had so many different types of exercise classes. We ended up trying to decide and ended up not doing any. This may inhibit people from engaging in physical activity (Mohammed- International student)*
**Beliefs about Capabilities** (Acceptance of the truth, reality or validity about an ability, talent or facility that a person can put to constructive use)	• Lack of the self-efficacy to engage in physical activity	√	√	*I can't do everything I want to do anymore because my lower joints won't let me (Catherine- Academic staff).*<br><br>*Um… to me I think number one, it's difficult. Why? Initially there used to be gym very close to my house at Moorways and they closed that place. Now which means I have to come to town, you know, and from my house to town will be about 30 minutes or 40 minutes to town. So, when I think about the time I have to walk or take the bus, and if I decide to stay back at uni, I don't normally come to uni every day, so as much as I would have loved to go to gym maybe two times in the week or three times, the distance to my house that's number one (Titilayo- International student).*

Sensitivity: Internal

**Environmental Context and Resources** (Any circumstance of a person’s situation or environment that discourages or encourages the development of skills and abilities, independence, social competence and adaptive behaviours).	• Barriers to physical activity opportunities in the university:<br>➢ Time constraints and timing	√	√	*Um… I don’t know, I guess like there are some sort of ventures, you know, if you wanted to go outside for a walk at lunchtime but like you say, they haven’t got much time to go far (Wendy- Administrative staff).*<br><br>*…it was 7.30 so of course people are being free, but most of the students are away so they’re not enjoying that, you could just watch or just try, so they are doing something but not at a good timing, so because of the timing… (Frank- PhD student)*
	➢ Financial constraints	√	√	*...but I’ve been down and it’s too expensive. I think they ought to give like a, I don’t know six weeks free or something or a month free just to encourage you. And then after that then you can decide whether it is worth the money what they’re asking. Because they do have a lot of classes down there, I think, don’t they? Because it is a big facility ain’t it? (Amy- Catering staff).*<br><br>*I wouldn’t be able to join a gym because I can’t afford gym membership and transport to a gym, I’d have to walk, like, an hour to get to the gym and then I’ve walked an hour, I’m not doing exercise on top of that (Martha- Master’s student).*
	➢ Work commitments	√	√	*I think you know, when you have a full-time job…I don’t have kids so…but still it feels like there is just always too many things to do in a day (Anita- Administrative staff)*<br><br>*Um… secondly, I’m… sometimes am heavily loaded with work and I’ll really want to go, like my colleagues usually go um… sometimes I want to go but I cannot because either am teaching or either I have to do something to meet some kind of deadlines (Ali- International student).*
	➢ Study commitments	-	√	*Like sometimes I’m in the uni till 10pm, 9pm, depending on the intensity of the work with deadlines for some publication and stuff like that. It takes so much time (Christopher- PhD students)*<br><br>*I think it’s similar to what you’ve said, like, the workloads just probably stopped me a little bit (Donald- First year undergraduate student).*
	➢ Family commitments	√	√	*Yeah, you know, yeah when got Jennifer has got children and when you’ve got children, kids always come first, don’t they? You’re canoeing is on the back burner ain’t it? (Anne- Catering staff).*<br><br>*…my research and time for family, so there is no time left (Christopher- PhD student).*
	➢ Weather	√	√	*And I think, I don’t know it is just, I think it is harder in winter to be healthier than in summer. I think you feel more like doing you know activities outside in summer. So, winter is a bad time but um… yeah, I think, I think everybody should do it (Amy- Catering staff)*<br><br>*The reason is this, back home in Nigeria, I used to trek from my house to the bus stop before getting a vehicle to work and then when I get to my bus stop to my workplace, I will trek again to my house… to the um… office. So, for me that was the form of exercise that I was engaging in,*

Sensitivity: Internal

				but when I got here the weather is so cold, so because the weather is cold, I'm not interested in going out. I just want curl up in bed (Oluchi- International student).
	➢ Inaccessibility to certain sports facilities.	√	√	*Um… If they had a swimming pool that would make a difference, perhaps um… that was a real missed opportunity in my opinion. Um… otherwise, I don't know, I think it would… it would be great to think that the university cared at all about our wellbeing, but I'm not holding my breath (Catherine- Academic staff).*<br><br>*They don't have like a way of having sports for all people, just for a few people, as Eric said, maybe 20 or 25% maximum students. If they want to increase, they have to think about completely again to what kind of facility they have and to access and, yeah (Frank- PhD students).*
	➢ Lack of advertisement	-	√	*I don't know, erm, maybe, like, where the lecture is will depend on how many stairs I climb. And, I suppose they could have more posters about what's available there, but I'm only at university two days a week and then the rest of it is me at home, so I don't think it really impacts that much (Martha- Master's student).*
	➢ High-rise buildings and location of university campuses	-	√	*Yeah, I think she's pretty right, I mean, you know, for me for example, I don't even take my lectures here, I take it at Friars Gate, which is just one long building, so it's a matter of getting to the building, jump in the lift, get to where you're going, come down, so there's really no room for that at all in Friars Gate (Bill- Master's student).*<br><br>*But I think, again, when you look at the whole, er, University as a whole itself, I, I don't really think so and that's nothing to do with the University of Derby, it's just the way it is, because I go to University of Nottingham a lot to use their library and because everything is just on one big, er, er, land mass, it's just you do a lot of walking and they've got these really beautiful footpaths and all that, so you see students doing a lot of walking, you know, from their Halls to classes, to the library, you know, erm, you do a lot of that, but obviously the University of Derby is scattered all over the place, so you have to jump on the bus, you have to, so I don't think the whole University of Derby take the way it is really, I'm not sure that's, er… yeah (Bill- Master's student).*
	➢ Social timetabling at the sport centre	√	-	*…as well and I think some of the sports…social timetables and things don't quite fit well for staff, they are a better fit for the students. And yeah, it's kind of geared a bit more for students than staff (James- Administrative staff).*
	➢ Free bus scheme	-	√	*Firstly, I don't think the university encouraging me for doing sport activity or just physical activity, but for another point of view, I noticed that Derby also does a free bus for students, it doesn't encourage them to walk, so they're just lazy (Frank- PhD student).*

Sensitivity: Internal

**Social/Professional Role and Identity** (a coherent set of behaviours and displayed personal qualities of an individual in a social or working setting)	• Difficulty in taking up opportunities to do physical activity	√	√	*There are so many opportunities in the university for us as administrative staff to be physically activity, but I guess the university authorities do not belief that it is important for us to be active. I feel they should have a timetable of activities at the sport centre that fit with our work routines. They focus more of students, which may be a major reason why most administrative staff are inactive (Peter- Administrative staff).*
	• Laziness to engage in physical activity	√	√	*I do not know about other academic staff but am very lazy when it comes to doing any form of physical activity. I guess most staff are too, with the ever-increasing workloads you barely have time for physical activity. You just want to get home and sleep (Monica- Academic staff).*<br><br>*I think like most administrative staff, I am very lazy with regards to engaging in physical activity. We are sat in front of our computers all day, so yes am pretty lazy when it comes to physical activity (Anita- Administrative staff)*<br><br>*Um… as I said before, I am very lazy when it comes to physical activity. I cannot say for other students, but I believe that students are generally lazy when it comes to engaging in physical activity (Oluchi- International student).*<br><br>*Yeah, I guess we are all lazy when it comes to physical activity. I mean the PhD students, including me. This may be due to our huge workloads, so at the end of the day we just want to hit our beds (Frank- PhD student).*
	• Physical activity not seen as an important attribute for university staff	√	-	*The university authority is not interested in encouraging the academic staff to be physically active. They just increase our workload almost on daily basis and do not really care if we engage in any form of physical activity. Since physical activity has been associated with many health benefits, as well as reduction in absenteeism from work, we should be given time to engage in some form of physical activity. However, this is not the case, as the university authority do not believe physical activity is important for us. That's my own opinion (Lynda- Academic staff).*
	• More focus on undergraduates	-	√	*The university only cares about the undergraduate students. I remember them having sports fairs in the atrium for the undergraduates to encourage them to be physically active. For us, the university does not care if we are active or not. I guess that is why most of us are inactive. There is no encouragement from the university to make PhD students physically active (Christopher- PhD students).*
**Optimism** (the confidence that things will happen for the best or that desired goals will be attained)	• Lack of confidence of the inactive to engage in physical activity	**-**	√	*I'm not very confident when it comes to swimming in deep sea and last time I experienced this, I lost my um… all confidence and I started to get panicked (Ali) , so when I came back I realised that I should do that, but again they are fully booked and there is no such trainers and we do not have much um… opportunities here. I mean… I find it really funny that council in Derby that they control all the swimming pools. That doesn't make sense to me, so I would like to have this set of skills (Ali- International student).*

Sensitivity: Internal

√= mentioned by at least one administrative staff or students

-= not mentioned by any administrative staff or student

NB: Pseudonyms were used for all the participants to protect their identity

Sensitivity: Internal
